# Continuous versus single shot adductor canal block for postoperative pain relief after total knee arthroplasty: A protocol for randomized controlled trial

**DOI:** 10.1097/MD.0000000000019918

**Published:** 2020-04-24

**Authors:** Yulin Tao, Qingjun Mao, Jixiang Wang

**Affiliations:** a Department of Anesthesiology; b Department of Orthopedics Centre; c Department of Critical Medicine, Sunshine Union Hospital, Shandong, China.

**Keywords:** adductor canal block, randomized controlled trial, study protocol, total knee arthroplasty

## Abstract

**Background:**

Adductor canal block has become a popular mode of pain management after total knee arthroplasty. This study compared a single-injection adductor canal block (SACB) with continuous adductor canal block (CACB). The hypothesis was that the 2 groups would have equivalent analgesia at 48 hours post-neural blockade.

**Methods:**

This is a double-blinded, randomized, controlled, equivalency trial that is conducted at a single university hospital in China. A total of 60 patients who meet inclusion criteria are randomized in a ratio of 1:1 to either CACB (0.5% ropivacaine 20 mL followed by continuous infusion of 0.2% ropivacaine at 5 mL/h for 48 hours) or SACB (0.5% ropivacaine 20 mL) group. The primary outcome is pain scores at 48 hours utilizing the visual analog scale, whereas the secondary outcomes include opioid consumption, Timed Up & Go test, ambulation distances at discharge, length of stay, and maximal flexion at discharge. All pain scores are assessed by an independent observer who is blinded to the allocation of groups.

**Results:**

This study has limited inclusion and exclusion criteria and a well-controlled intervention. This clinical trial is expected to provide evidence of better therapy for the pain management after total knee arthroplasty.

**Trial registration:**

This study protocol was registered in Research Registry (researchregistry5431).

## 1 Introduction

Currently, total knee arthroplasty (TKA) has been widely used in treatment for the elderly patients with end-stage osteoarthritis.^[1,2]^ However, due to the soft tissue injury and large amount of bone destruction involved, undesirable postoperative pain remains a challenge for both patients and surgeons after TKA.^[3]^ Previous study has shown that 23% of patients cite at home pain as “severe/extreme” after surgery, whereas 54% of the patients indicate “severe pain at least some of the time.”^[4]^ In addition, it has been suggested that severe pain can also interfere with the recovery process, which increases the risk of postoperative complications, including infection, joint loosening, and reflex sympathetic dystrophy.^[5]^

At present, various techniques can be used to relieve postoperative pain, including epidural anesthesia, femoral nerve block, adductor canal block (ACB), and local infiltration analgesia. While femoral nerve block has traditionally been the gold standard for analgesia following TKA, it significantly impairs quadriceps motor function, which may interfere with rehabilitation and delay discharge.^[6,7]^ Recently, ACB has emerged as an alternative to femoral nerve block, with the advantage of sparing the motor nerve supply to most of the quadriceps muscle and may lead to a reduction in falls after surgery.^[8,9]^ However, the optimal duration to maintain ACB is unknown. Some hospitals use a single shot adductor canal block (SACB), while others use a continuous catheter to maintain the infusion for 24 hours or 48 hours after surgery. The advantages of continuous infusion over a single injection are debatable. Critics argue that similar analgesia can be achieved with SACB, especially as the duration of the single-shot block can be extended over 12 hours in some patients, while the insertion and maintenance of continuous catheters is resource- and labor-intensive. Another argument against continuous infusion is that longer blocks may adversely affect physiotherapy and delay patient rehabilitation after surgery.^[9,10]^

However, limited randomized clinical trials have been conducted to compare the efficacy of SACB with continuous adductor canal block (CACB) in patients undergoing TKA. The aim of the current study was to compare SACB and CACB techniques with regard to early period pain levels, need for additional opioids, and ambulation and functional scores in patients who had undergone primary TKA. We hypothesized that there was a significant difference between the CACB group and the SACB group in terms of postoperative analgesia, opioids consumption, ambulation ability, and early functional recovery after TKA.

## 2 Materials and methods

This study was a prospective randomized blinded study, with a parallel design and an allocation ratio of 1:1 for the treatment groups. The study was approved by the Institutional Review Board in our hospital (PS2020037) and was registered in the Research Registry (researchregistry5431). Written informed consent was obtained from all subjects prior to enrolment. This study was performed and reported in accordance with the principles of the Declaration of Helsinki (2000).

### 2.1 Patients

The inclusion criteria were set as follows: osteoarthritis of the knee requiring primary TKA during hospitalization; patients that were over 18 years old and could cooperate with us for treatment and postoperative observation; American Society of Anesthesiologists status of I to III. Exclusion criteria included patients with a body mass index of ≥40 kg/m^2^ and allergy to local anesthetics, systemic opioids (fentanyl, morphine, hydromorphone), or any of the drugs included in the multimodal perioperative pain protocol. We also excluded patients undergoing revision knee arthroplasty, those with impaired kidney function or a coagulopathy, and those with chronic pain syndromes or chronic opioid use. Chronic pain was defined as use of regular daily doses of systemic opioids in the 6 months prior to the surgery.

### 2.2 Randomization

**Figure 1:**
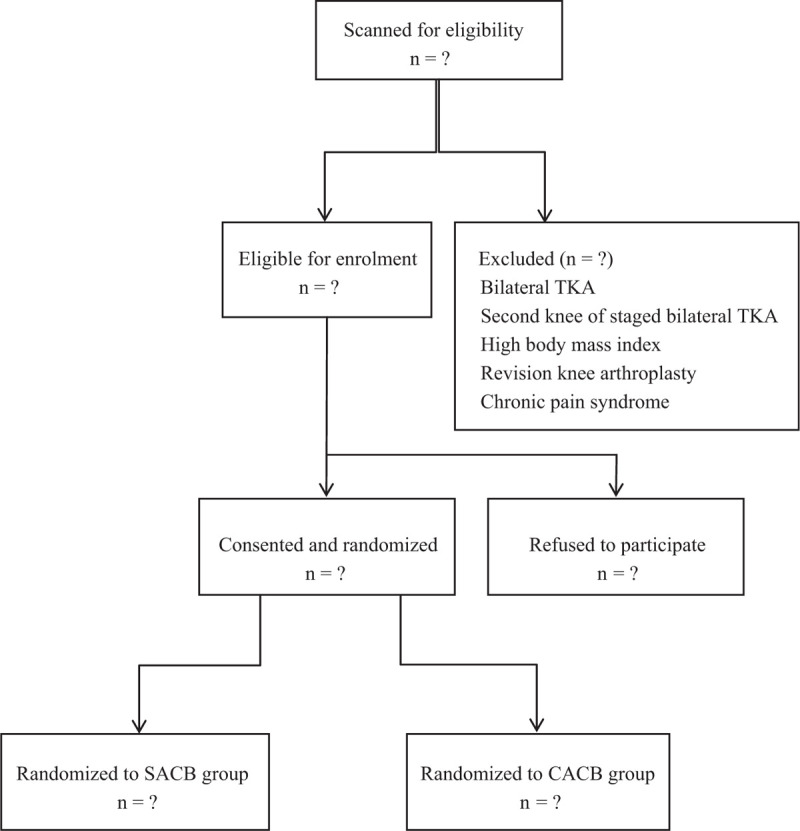
Flow diagram of the study.

A computer-generated randomization table was used for patient allocation to one of the 2 study groups: the SACB group or the CACB group. Each time a patient is included in the trial, the generated randomized number is assigned accordingly. The patients assigned an even number are allocated to the CACB group and those with an odd number are allocated to the SACB group. Patients’ assignments were written in a sealed envelope, which was only opened after the patient had consented to the study (Fig. 1).

### 2.3 Interventions

#### 2.3.1 SACB

The ACB was performed while the patient remained under spinal anesthesia. A 13 to 6 MHz linear transducer on a SonoSite Edge ultrasound machine (SonoSite, Bothell, WA) was used to perform an anatomical survey of the medial thigh with identification of the femoral artery, sartorius and vastus medialis muscles. A 22G × 80 mm SonoPlex STIM needle (Pajunk, Geisingen, Germany) was introduced in plane lateral to medial and 20 mL of 0.5% ropivacaine was deposited at the midportion of the adductor canal with peri-arterial spread around the femoral artery as the end point.

#### 2.3.2 CACB

Under ultrasound guidance as above, a 17G Tuohy needle (Arrow StimuCath, Reading, PA) was introduced into the adductor canal. Following dilation of the adductor canal with normal saline, a 19G nerve catheter was threaded up to 3 to 5 cm beyond the needle tip. The guidewire was removed upon the catheter exiting the needle tip while threading to avoid inadvertent advancement of the catheter out of the space. The catheter was then manipulated and normal saline injected to confirm the catheter tip location within the adductor canal on ultrasound visualization, with peri-arterial spread as the endpoint. Up to 5 to 10 mL of normal saline was used in total per catheter placement. Twenty milliliter of 0.5% ropivacaine was then injected via the catheter, following which a continuous infusion of 0.2% ropivacaine commenced at 5 mL/h for 48 hours and then removed. On the ward, the infusion could be temporarily stopped to facilitate physiotherapy or mobilization with prompt resumption post activity. If the catheter failed, was replaced, or fell out after surgery, patients were withdrawn from the study.

### 2.4 Postoperative care

Postoperative drainage lasted 1 to 2 days until flow volume was <30 mL. All patients received the same standardized postoperative multimodal pain protocol, with 4 doses of 1 g of acetaminophen, 2 doses of celecoxib 200 mg, and morphine (first 48 hours) or tramadol (after 48 hours) for pain exacerbations. All patients underwent the same postoperative rehabilitation program, with partial weight bearing with the use of crutches for the first postoperative day and active range of movement exercises.

### 2.5 Outcome evaluation

At the time of admission, patients were explained about the visual analog scale, pain scale, and mobilization ability assessment. Patients were assessed for pain at 4, 8, 12, and 24 hours postoperatively, pain at rest, pain after mobilization on POD1 and POD2, opioid consumption, side effects if any. Ambulation ability was assessed 24 hours after the block, in form of Timed Up & Go (TUG) test, 10 m walk test, and 30 seconds chair stand test. Furthermore, ambulation distances at discharge, maximal flexion at discharge, and length of hospital stay were evaluated. Pain was evaluated on a VAS with 0 = no pain, and 100 = worst imaginable pain. The TUG test measures the time it takes a person to stand up from a chair, walk a distance of 3 m, and return to the chair. The 10-m walk test measures the time it takes to walk a distance of 10 m as quickly as possible. The 30-second Chair Stand test assesses how many times a person is able to rise from a chair and sit down again in 30 seconds, with the arms kept crossed over the chest. During the assessment of ambulation ability, use of gait aids was not allowed. The tests were only performed if the subject felt that it was possible without the risk of falling.

### 2.6 Statistical analysis

Statistical analyses were conducted using SPSS v22.0 software (IBM, Chicago, IL). Conformity of the data to normal distribution was tested with the Kolmogorove–Smirnov test. Independent 2 samples *t* test was used for comparison of continuous variables and Pearson chi-square test was used for comparison of categorical variables. Results were evaluated in a confidence interval of 95% and at a significance level of *P* < .05.

### 2.7 Power analysis

The trial was designed to test equivalency between groups. Using an 11-point numerical pain scale (0–10), the study was powered to find that the 2 approaches to nerve blockade do not differ by >2-points on the pain scale (the assumption being that a difference of <2 numerical points on the pain scale is not clinically significant). We used an alpha value of 0.05 and a power of 90%. Preliminary data from 50 subjects with a CACB following TKA at our institution showed a mean movement pain score at 36 hours of 5.08 and a standard deviation of 1.94. Power analysis determined that 26 patients in each group would be needed. Allowing for 10% dropout, 30 patients per group (60 total patients) were enrolled in the study.

## 3 Discussion

ACB, an alternative form of peripheral nerve block, is almost a pure sensory nerve block. Several studies, in recent past, had reported the efficacy of ACB in management of analgesia following TKA.^[4–6]^ Moreover, few studies had demonstrated the superiority of ACB in preserving quadriceps muscle strength and thereby early mobilization compared with Femoral nerve block (FNB).^[6–8]^ In addition, the administration of ACB may be accomplished either as a single shot injection or as a continuous block using epidural catheter and infusion. Furthermore, few studies in literature has studied the differences in efficacy of a SACB or CACB post-TKA.^[11–13]^ Hence, an ideal regimen for adductor canal blockade to provide optimum pain relief and concomitantly promote early patient mobilization following TKA needs to be defined.

This trial has some limitations. First, the subjects may be exclusively Chinese. Therefore, the data from this clinical trial cannot be applied to other ethnic groups. Second, owing to the small sample size, the results of this study cannot be generalized. Despite these limitations, this trial is expected to provide evidence of better therapy for the pain management after total knee arthroplasty.

## Author contributions

Yulin Tao planned the study design and wrote the study protocol. Yulin Tao, Qingjun Mao, and Jixiang Wang reviewed the study protocol. Yulin Tao and Qingjun Mao will recruit participants and collect data. Yulin Tao wrote the manuscript. All of the authors have read, commented on, and contributed to the submitted manuscript.

Jixiang Wang orcid: 0000-0002-8201-4515.
